# Do the benefits of polyandry scale with outbreeding?

**DOI:** 10.1093/beheco/arv103

**Published:** 2015-07-01

**Authors:** Emily R. Burdfield-Steel, Sam Auty, David M. Shuker

**Affiliations:** ^a^School of Biology, University of St Andrews, Harold Mitchell Building, St Andrews, Fife KY16 9TH, UK and; ^b^Department of Biological and Environmental Science, Centre of Excellence in Biological Interactions, University of Jyväskylä, PO Box 35, Jyväskylä 40014, Finland

**Keywords:** genetic compatability, polyandry, reproductive interference, sexual selection.

## Abstract

There have been many potential explanations put forward as to why polyandry often persists despite the multiple costs it can inflict on females. One such explanation is avoidance of costs associated with mating with genetically incompatible males. Genetic incompatibility can be thought of as a spectrum from individuals that are genetically too similar (inbreeding) to those that are too dissimilar (outbreeding or hybridization). Here we look for evidence that the level of outbreeding influences the benefits of polyandry in the seed bug *Lygaeus equestris*. Our system allows us to test for benefits of polyandry at levels of genetic similarity ranging from full siblings to heterospecifics, both in terms of egg production and hatching success. We found that while outbreeding level appeared to have no effect on fitness for intraspecific matings, and polyandry did not appear to result in any increase in fertility or fecundity, hybridization with a closely related species, *Lygaeus simulans*, carried considerable fitness costs. However, these costs could be rescued with a single mating to a conspecific. Thus, polyandry may be beneficial in populations that co-occur with closely related species and where there is reproductive interference. However, within-species genetic incompatibility is unlikely to be the driving force behind polyandry in this species. Furthermore, the mechanism underlying this rescue of fertility remains unclear as manipulation of male cuticular hydrocarbon profile, a possible mechanism by which females can assess male identity, had no effect on female offspring production.

## Lay Summary

Mating can be both costly and dangerous. Despite this, females of many species typically mate more than once and with different males. We found that female seed bugs only benefit from mating with more than 1 male if one of her mates was a different species. Females needed only 1 mating with her own species to maximize her fitness. Thus, multiple mating may be adaptive in areas where the 2 species coexist.

## INTRODUCTION

Mating is known to carry nontrivial costs for females, yet female polyandry is widespread in insects (Arnqvist and Nilsson 2000; Hosken and Stockley 2003; Pizzari and Wedell 2013; Simmons 2005). Traditionally, female reproductive success has been viewed as not depending on the number of copulations they have (Bateman 1948), which makes female multiple mating a puzzle (Arnqvist and Nilsson 2000). Potential explanations for female polyandry can be broadly categorized under several headings (Boulton and Shuker 2013). Firstly, female mating rate may be the result of conflict between the sexes over mating, shifting mating rate away from the (presumably low) female optima (Chapman et al. 2003). One outcome of this is convenience polyandry, where females mate to mitigate the costs of harassment by males (good examples include water striders: Rowe et al. 1994 and seaweed flies: Shuker and Day 2001; Thornhill and Alcock 1983). Secondly, females may receive benefits from mating more than once, which can be classified as either direct or indirect benefits.

Direct benefits increase the fitness of the female by increasing investment in offspring production. For instance, access to food gifts during courtship and mating (Fedorka and Mousseau 2002; Gwynne 2008) or increased male parental care or protection (Ihara 2002) are clear benefits to polyandrous females. However, there are numerous species where nuptial feeding and paternal care are nonexistent, yet polyandry still occurs (Arnqvist and Nilsson 2000). Multiple mating may also ensure full fertility though, for instance if a single copulation involves the transfer of insufficient sperm (Pai et al. 2005; Wang and Davis 2006). Nonetheless, some studies have shown that females may benefit more from mating with different males, as opposed to just one male multiple times (Newcomer et al. 1999). For example, in the pseudoscorpion *Cordylochernes scorpioides*, females that received a spermatophore from 2 different males produced 32% more offspring than those that received 2 from a single male (Newcomer et al. 1999). Similarly, in the field cricket (*Gryllus bimaculatus*), hatching success increased with increasing number of mates (Tregenza and Wedell 1998). This suggests an alternative source of selection favoring polyandry: indirect benefits.

Indirect benefits are those that increase offspring fitness, that is, through genetic mechanisms. These include higher quality paternal genes, that is, “good genes” or “sexy sons” (Jennions and Petrie 2000; Byrne and Rice 2005), as well as increased genetic diversity or genetic compatibility. Thus, female polyandry could be favored if it allows the acquisition of such indirect (i.e., genetic) benefits. In the case of increased genetic diversity, females may benefit from producing offspring of diverse genetic make-up (Mattila and Seeley 2007), both to insure against future environmental perturbation and to potentially reduce sibling competition. Additionally, mating with multiple males might allow females to avoid the costs of genetically incompatible sperm (Jennions and Petrie 2000; Tregenza and Wedell 2000; Tregenza and Wedell 2002). In the case of good genes, all females should agree on which males carry good genes (i.e., genes currently favored by natural selection). However, a number of studies have shown that offspring fitness depends on specific male–female combinations (Tregenza and Wedell 1998; Agbali et al. 2010). This provides strong support for the genetic incompatibility avoidance hypothesis, where a male’s suitability depends on the genome of the female.

Genetic incompatibility can be viewed as a spectrum, arising from inbreeding depression at one end, through to genetic incompatibility between divergent gene pools following reproductive isolation at the other (often viewed in the framework of Dobzhansky–Muller incompatibilities: Orr and Turelli 2001; see Shuker et al. 2005 for an empirical example). Polyandry could therefore evolve to avoid incompatibilities arising from both inbreeding depression, that is, mating with close relatives, and outbreeding depression, that is, across a species barrier, for example, mating with a sister species (Arnqvist and Nilsson 2000). There is evidence in several systems for homogamy, or a bias for conspecific sperm (Fricke and Arnqvist 2004; Kyogoku and Nishida 2013), which could allow polyandrous females to avoid the costs associated with mating with a heterospecific. If this is the case then we would expect polyandrous females to possess mechanisms to bias fertilization toward compatible males (Bretman et al. 2004).

Females of the seed bug *Lygaeus equestris*, and the closely related species *Lygaeus simulans*, show high rates of infertility (oviposition of unfertilized eggs), presumably as a result of mating failure (Greenway and Shuker forthcoming). Although mating failure has been documented in many insects (Eberhard 1996; Garcia-Gonzalez 2004), it has only recently been receiving theoretical attention (Rhainds 2010; see also Hasson and Stone 2009). Mating failure is often viewed in the context of females failing to mate, presumably due to lack of opportunity. Less is known about situations in which females mate, but these matings do not lead to the production of offspring (“cryptic mating failure”: Greenway et al. forthcoming). The promiscuous mating system of *L. equestris*, whereby both males and females mate with multiple individuals, could have evolved in response to this high risk of mating failure. Postmating infertility has been demonstrated in several species of Lygaeidae (McLain 1991; Tadler et al. 1999). The exact mechanism behind these mating failures is unknown, but there are a number of potential explanations. For instance, it may be the result of failure by the male to successfully inseminate the female during copulation (all species reported to show mating failure have complex genitalia, and a minimum copulation duration of 1h is necessary for sperm transfer: Micholitsch et al. 2000; Higgins et al. 2009; Dougherty and Shuker 2014). Additionally, as many laboratory pairings are performed with virgins, there could be an effect of first matings, as virgin males may be less efficient at achieving insemination or virgin females may be less willing to accept their first mates (but see Greenway and Shuker forthcoming for evidence of the repeatability of mating failure in *L*. *simulans*).

In our first experiment, we explored the possible benefits of polyandry in the face of variation in the level of outbreeding, from full-sibs through to heterospecific matings (*L. equestris* and *L. simulans* are capable of hybridizing, including the production of F2 offspring, although a detailed analysis of hybrid fitness remains to be done: Evans et al. forthcoming; see Bramer et al. (2015) for a recent phylogeny of the Lygaeidae, including these 2 species). We used nonvirgin males and gave all females 2 mating opportunities in order to reduce the chances that the level of mating failure recorded was due to male or female sexual inexperience. We took advantage of the multiple levels of genetic distance available in the laboratory to test the effect of different levels of inbreeding and outbreeding on the possible benefits of polyandry in *L. equestris*. We expect to see greater benefits at the 2 extremes of the genetic range, that is, in the inbreeding full-sibling treatment, and also in the outbreeding treatment, when females are mated to *L. simulans* males, as this is where genetic incompatibilities are most likely to occur.

In our second experiment, we focused on the cost of hybridization when female *L. equestris* are paired with male *L. simulans* versus conspecific males. In particular, we looked at whether fecundity could be rescued by differential fertilization (i.e., homogamy), and if mating order (conspecific followed by heterospecific and vice versa) had any effect. If females can indeed bias sperm use toward conspecific sperm, we would expect to see comparable rates of rescue regardless of mate order. Alternatively, sperm may be used equally, providing only a partial rescue of fertility. Finally, a last male advantage may occur, allowing for fitness to be rescued only if the female’s last mate was a conspecific. Our third experiment follows the same design as experiment 2, but each male was washed with hexane in order to disrupt his cuticular hydrocarbon (CHC) profile. Recent work (Burdfield-Steel ER, unpublished data) suggests that these CHCs may play a role in mate choice and species discrimination in *Lygaeus*; hence, removal may limit the female’s ability to bias fertilization toward conspecifics.

## METHODS

For this study, we utilized 2 populations of *L. equestris*, and a population of *L. simulans*. The Dolomites population of *L. equestris* originates from bugs collected in the Dolomites mountains in northern Italy in 2004 by David Shuker and colleagues. The Leeds population of *L. equestris* was collected in Sicily and then maintained in laboratory culture in Sweden before a new laboratory culture was founded at the University of Leeds by Professor Nina Wedell in 1996. The *L. simulans* population was collected in Tuscany, Italy in 2006, also by Shuker and colleagues. We isolated 5th instar *L. equestris* nymphs (from the Dolomites population) and housed them in tubes with food and water provided. Nymphs were checked daily and newly eclosed adults were removed, and housed in single-sex tubs with no more than 10 individuals. *L. equestris* typically become sexually receptive between 5 and 6 days after eclosion, therefore all adults used in this experiment were a minimum of 7 days old to ensure they were sexually mature. Seven days after eclosion, virgin females were assigned to a male and housed with him for 24h to allow mating. This time period was chosen as prolonged struggles often occur of over mating in this species (personal observation), and it may take several hours for successful insemination to occur once mating has begun. Males were then removed and females left to oviposit. A single clutch was collected from each female in order to create full-sib cohorts. When the nymphs from these cohorts reach 5th instar they were checked daily and newly eclosed adults removed and house in single-sex groups of siblings. Males from the Leeds population of *L. equestris* and *L. simulans* males were isolated from continuous culture as 5th instar nymphs and after eclosion were kept in single-sex tubs of no more than 10 individuals. At 7 days virgin males from each cohort, as well as those from the other population and species, were assigned to a single (nonsibling) female from the same population and housed with her for 2h to allow mating. They were then kept in male only groups, with either siblings or males from the same population, for 24h prior to the start of the experiment. The females were discarded.

### Experiment 1

At the start of experiment 1, females from the same clutch were assigned to one of the 8 treatments. In treatment 1, females were twice given the opportunity to mate with a (once mated) male from the same clutch. In treatment 2, females were twice given the opportunity to mate with a male from the same population but a different clutch. In treatment 3, females were twice given the opportunity to mate with a male from the other population of *L. equestris*, and in treatment 4, females were twice given the opportunity to mate with a *L. simulans* male. Treatments 5–8 follow the same pattern but rather than being given the opportunity to mate with the same male twice, females were instead given the opportunity to mate with 2 different males (see Supplementary Figure 1 for a graphical representation of the experimental design). The second male had previously been housed with a different female in the same treatment. All males in the experiment had the opportunity to mate twice during the course of the experiment in addition to their 1 mating prior, those that were in treatments 5–8 had the opportunity to mate with 2 different females while those in treatments 1–4 had the opportunity to mate with the same female twice. In all cases, females were housed for 24h with a male, and then isolated for 24h before being housed either with the same or a different male for a further 24h. Thus, males had 24h between matings to replenish sperm reserves.

After mating females were housed individually in tubs until death. The number of eggs laid was checked daily and if eggs were present the female was moved to a new tub. All eggs were then returned to the incubator for a further 10 days after which the number of nymphs present was recorded as a measure of hatching success. Eggs were collected from a total of 306 females, with sample sizes per treatment ranging from 31 to 50. The effect of number of mates and level of inbreeding on both nymph and egg production was analyzed in R version 2.15.1. Linear mixed models were fitted with family as a random factor. Age at death was also included in the model as lifespan, and egg and nymph production were correlated. The influence of both factors on the rate of mating failure (defined as a female producing no nymphs in the course of her life) was modeled with a binary logistic generalized linear model (GLM) in PASW Statistics 18 by IBM.

### Experiments 2 and 3

Experiment 2 consisted of 4 treatments. *L. equestris* females were mated to each of the following: either 2 *L. equestris* males, 2 *L. simulans* males, an *L. simulans* male followed by an *L. equestris* male or an *L. equestris* male, followed by an *L. simulans* male. For experiment 3, the same experimental treatments were conducted, but all the males were washed with hexane to reduce their CHCs. Care was taken while handling the males to avoid any damage. The hexane was applied using 2 paintbrush strokes along the dorsal surface of the males (see Supplementary Figure 2 for a graphical representation of the design of both experiments). Hexane, an organic solvent, is commonly used in the removal of hydrocarbons and the technique is relatively widespread (Tregenza and Wedell 1997; Burdfield-Steel 2014). In unpublished work by Burdfield-Steel et al. on *Lygaeus* seed bugs, on removal of CHCs no gross difference in behavior was observed and mating activity did not differ significantly (Burdfield-Steel 2014).

The trials were performed in blocks to obtain suitable sample sizes (*n* = 30 for each treatment). Within each block, every effort was made to have equal numbers for each treatment. Only adults that were at least 7 days old after eclosion were used to ensure sexual maturity. Each female was placed in a small tub with a water tube and some sun flower seeds, and either an *L. equestris* or *L. simulans* male was then introduced. As in experiment 1, each pair was given 24h for mating to take place and any pairs still copulating at the end of this period were separated using a paintbrush. The females were then given a rest day before being paired with the second male for another 24h. Trials where either the female or male died during the matings were discounted. Once the second males were removed, egg production was recorded daily. As data from experiment 1 showed that significant changes in egg and nymph production occurred within the first 7 days following the final mating, egg production for each female was only recorded over this period. New males were used for each block of trials to negate any possible bias through increased male mating experience. Egg and nymphs were counted as in experiment 1. Any females that died during the 7-day egg-laying period were recorded as doing so. In total, 240 females were studied, with 30 females in each treatment.

Egg and nymph numbers were then summed for each female, and the mean and standard error were calculated for each experimental treatment. Anova and post hoc analysis (Tukey’s Honestly Significant Difference test) were then used to test for significant differences between each treatment. In addition, the mean hatching success for each treatment was calculated by dividing nymph production by egg production; however, for this analysis, females that laid no eggs throughout the 7-day period were removed to avoid artificially biasing the proportion of eggs hatched. Chi-square tests were used to test for differences between treatments in the number of females producing no eggs and no nymphs.

## RESULTS

### Experiment 1

**Figure 1:**
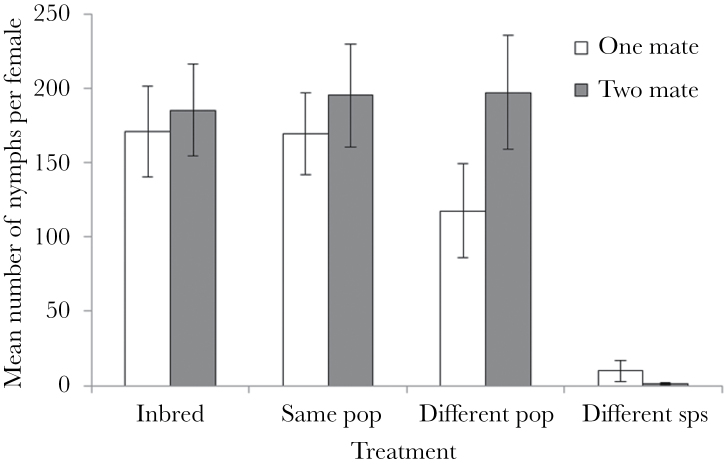
The mean number of nymphs produced per female across the treatments in experiment 1. Females were given the opportunity to mate twice either with the same male or with 2 different males, from one of the 4 levels of outbreeding. Error bars indicate ±1 standard error.

**Figure 2:**
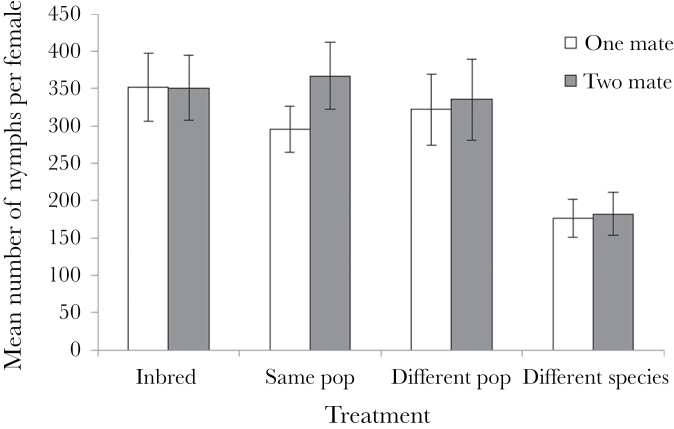
The mean number of eggs produced per female across the treatments in experiment 1. Females were given the opportunity to mate twice with the same male either or with 2 different males, from one of the 4 levels of outbreeding. Error bars indicate ±1 standard error.

**Figure 3:**
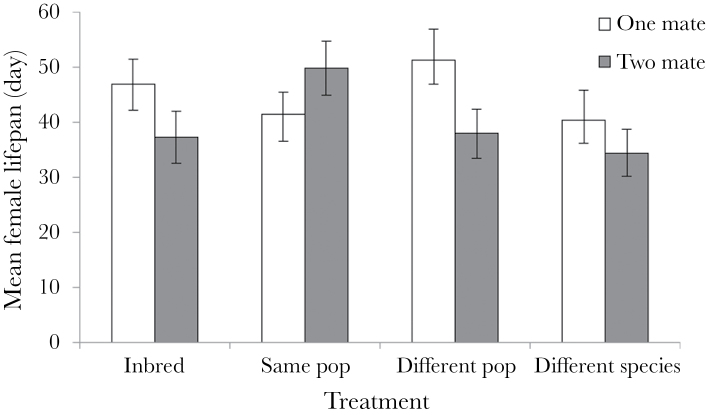
The mean lifespan of females across the treatments in experiment 1. Females were given the opportunity to mate twice either with the same male or with 2 different males, from one of the 4 levels of outbreeding. Error bars indicate ±1 standard error.

**Figure 4:**
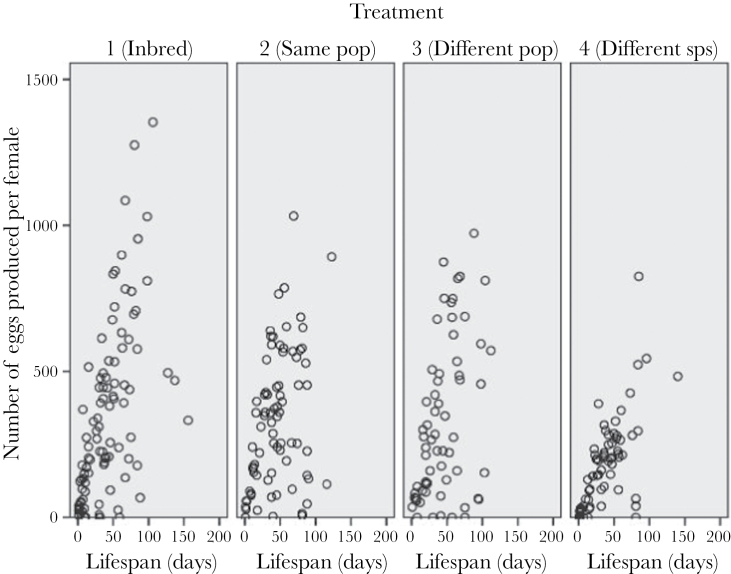
Female egg production as a function of lifespan for each of the 4 levels of outbreeding.

**Figure 5:**
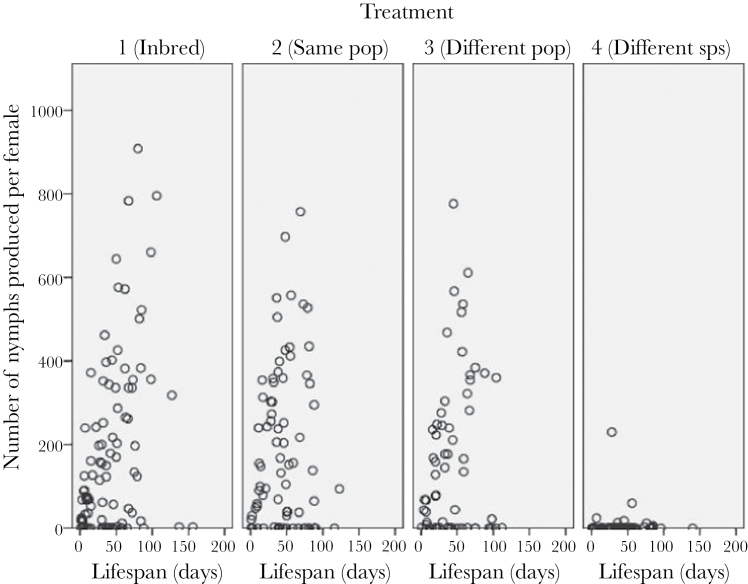
Female nymph production as a function of lifespan for each of the 4 levels of outbreeding.

Female fitness was influenced by outbreeding level, but not by the number of mates she had. Mate number did not significantly affect the number of nymphs (*F*_1, 305_ = 2.24, *P* = 0.14) or eggs (*F*_1, 305_ = 0.89, *P* = 0.35) a female produced. However, both measures were affected by outbreeding level (*F*_3, 303_ = 22.27, *P* < 0.0001 and *F*_3, 303_ = 11.21, *P* < 0.0001 for nymph and egg number, respectively), and this seems to be a result of lowered egg and nymph production in females mated to *L. simulans* males (Figures 1 and 2). Unsurprisingly, there was a significant association with female lifespan (*F*_1, 305_ = 37.45, *P* < 0.0001 and *F*_1, 305_ = 163.85, *P* < 0.0001 for nymph and egg number, respectively) as, on average, females who lived longer produced more eggs. Female lifespan was affected by the number of mates she had however (*F*_1, 305_ = 5.41, *P* < 0.05), as females mated to 2 different males had lower average lifespans (Figure 3). Despite this apparent difference between the treatments, the interaction between number of mates and outbreeding level was not significant and outbreeding level did not influence lifespan. However, there was a significant interaction effect between lifespan and outbreeding level (*F*_3, 303_ = 7.90, *P* < 0.0001 and *F*_3, 303_ = 5.55, *P* < 0.01 for nymph and egg number, respectively). Females mated to conspecifics showed greater gains in both egg and nymph production for each extra day they lived in comparison to females mated with *L. simulans* males (Figures 4 and 5). All other interactions were nonsignificant (*P* > 0.05).

If the *L. simulans* treatment was excluded from the analysis of nymph number, then outbreeding level no longer significantly influenced number of nymphs produced (*F*_2, 234_ = 0.10, *P* = 0.904), nor did number of mates (*F*_1, 235_ = 2.744, *P* = 0.098). Lifespan and the interaction between lifespan and outbreeding level both remained significant (*F*_1, 235_ = 37.94, *P* < 0.0001 and *F*_2, 234_ = 4.36, *P* = 0.014, respectively). All other interactions were nonsignificant.

Outbreeding level did have a significant effect on the probability of mating failure (χ^2^ = 56.94, degrees of freedom [df] = 3, *P* < 0.0001); however, the number of mates did not (χ^2^ = 0.98, df = 1, *P* = 0.324). Nor did the interaction between the two (χ^2^ = 4.71, df = 3, *P* = 0.195). As with nymph number, this effect was driven by the high levels of infertility in the *L. simulans* treatment.

### Experiment 2

**Figure 6:**
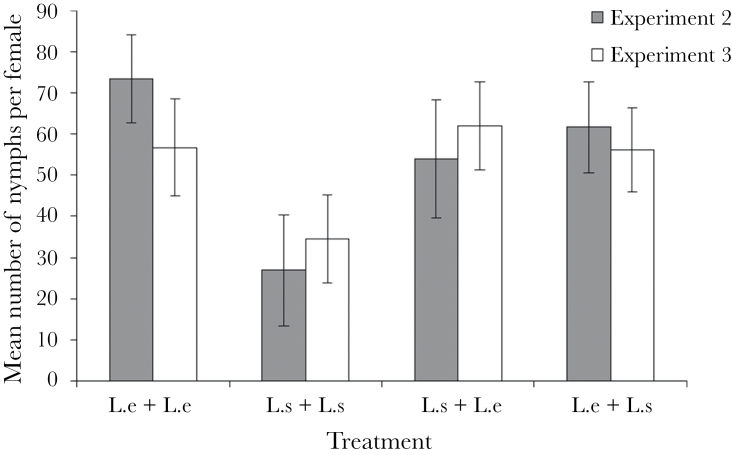
Mean number of nymphs produced per female across each experimental treatment in experiments 2 and 3. In experiment 2, females were mated with 2 unmanipulated males of the corresponding species combination, whereas in experiment 3, the males were washed with hexane prior to being introduced to the female. Treatment 2 (*L. simulans* + *L. simulans*) had significantly lower nymph production in experiment 2. *N* = 30 for each treatment. Error bars indicate ±1 standard error.

Mating with a conspecific rescued offspring production for female *L*. *equestris* when also paired with a heterospecific. In terms of egg production, females laid similar numbers of eggs regardless of mating with conspecifics, heterospecifics, or one or both (Anova: *F*_3,116_ = 0.76, *P* = 0.52; Figure 6). This suggests that female *L*. *equestris* do not need to mate with a conspecific to initiate oviposition. Overall, 89% of females produced eggs. The number of females producing no eggs differed significantly with treatment, with 9 out of the 13 cases occurring when a female mated first with a heterospecific and then with a conspecific male (chi-square test: χ^2^ = 16.82, df = 3, *P* < 0.001).

**Figure 7:**
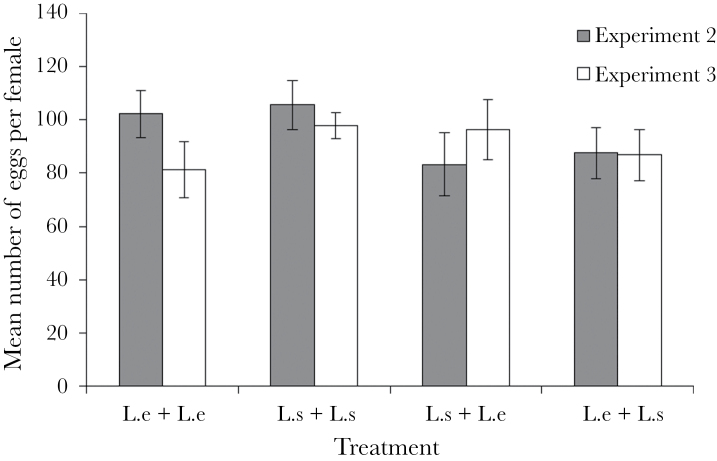
Mean number of eggs laid per female across each experimental treatment in experiments 2 and 3. In experiment 2, females were mated with 2 unmanipulated males of the corresponding species combination, whereas in experiment 3, the males were washed with hexane prior to being introduced to the female. Egg production did not differ significantly with treatment. *N* = 30 for each treatment. Error bars indicate ±1 standard error.

In terms of offspring production, there were however large differences between the treatments in terms of the number of nymphs produced (*F*_3, 116_ = 4.91, *P* = 0.003; Figure 7). Females mated to 2 heterospecifics produced the fewest nymphs, and significantly fewer than females in the other 3 treatments (Least Significant Difference [LSD]: all *P* < 0.033). This means that mating with just one *L. equestris* male appeared to rescue full fertility in terms of nymph production, and it did not appear to matter whether *L*. *equestris* females mated first or second with a conspecific for this rescue effect to occur (Figure 7). However, while mean nymph production did not show any order effects, the number of females producing no nymphs differed significantly with treatment as before, with 12 out of the 21 females being in the treatment in which a heterospecific mating was followed by a conspecific mating (χ^2^ = 14.03, df = 3, *P* = 0.003).

In keeping with the nymph production result, the proportion of eggs hatched significantly differed across treatments (binomial GLM: *F*_3,103_ = 23.78, *P* < 0.0001), with females mating to with heterospecifics having significantly lower hatching rates than females in the other 3 treatments (LSD: all *P* < 0.001).

### Experiment 3

As in the previous experiment, the number of eggs laid did not differ significantly between treatments (*F*_3, 116_ = 0.52, *P* = 0.67; Figure 7). In terms of nymph production and hatching success, similar overall patterns were found as before (Figure 6); however, in this experiment, the difference between treatments was found not to be significant for nymph production (*F*_3, 116_ = 1.71, *P* = 0.17) while it still was for hatching success (binomial GLM: *F*_3, 101_ = 7.88, *P* < 0.0001; treatment 2 different to all others, pairwise LSD: *P* < 0.001). About 87.5% of females laid eggs, whereas 65.8% gave rise to at least 1 nymph. Across treatments, there was no significant difference in number of females laying no eggs (χ^2^ = 3.28, df = 3, *P* = 0.35) or no nymphs (χ^2^ = 0.41, df = 3, *P* = 0.94).

## DISCUSSION

Understanding the evolutionary origin and maintenance of polyandry remains a central issue in behavioral ecology, relevant both to understanding the evolution of mating systems, and also the action of sexual selection (e.g., Shuker and Simmons 2014). Here we have explored the extent to which different levels of outbreeding influence the costs and benefits of female multiple mating in *L. equestris*, a highly polygynandrous insect. The level of outbreeding within *L. equestris* had no effect on either the number of eggs or nymphs produced by females. There was an effect across the whole inbreeding–outbreeding spectrum; however, this was driven by the low fitness of the *L. equestris × L. simulans* crosses (i.e., the cost of hybridization). Our results are perhaps a little surprising given that inbreeding depression, in the form of a reduction in fertile eggs, has been described in other populations of *L. equestris* (Laukkanen 2014) and that mating failure appears to be rather common in our populations. However, it is possible that prolonged laboratory culture has purged this populations of deleterious recessive alleles that contribute to inbreeding depression (Tregenza and Wedell 2000). Alternatively, the costs of inbreeding may be expressed later in development, as our design measured only the number of eggs laid, and hatching success of those eggs.

Furthermore, we found no effect of polyandry on female fitness, regardless of her level of relatedness to her mates. There was some suggestion that multiple mates may reduce female lifespan, but this did not appear to significantly impact egg or nymph production. Although it appears that nymph production was higher when females were mated to 2 males from a different population of the same species rather than one (see Figure 1), this effect was not significant. Our data therefore do not support the idea that polyandry has evolved or is maintained in *L*. *equestris* as a way to ensure genetic compatibility, although given our lack of inbreeding effects that is perhaps not surprising, as inbreeding has been suggested to be the most plausible basis of genetic incompatibility. While our results are in contrast to the findings in *G. bimaculatus* (Tregenza and Wedell 2002), a review of the polyandry literature in 2005 found that, while there generally was a positive effect of polyandry on hatching success, the effect size was small, and that pattern did not hold true for all systems (Simmons 2005). A meta-analysis in 2012 found similar results, with polyandry having a positive effect on clutch production and fertility, as well as potentially hatching success, but that these effects were weak (Slatyer et al. 2012). Thus, with the exception of heterospecific crosses, female polyandry in our populations is unlikely to be favored by any increased fertilization success resulting from the avoidance of incompatible sperm. Instead, female multiple mating in this system may be favored by a number of alternative processes, including the risk of sperm depletion (Wang and Davis 2006) or perhaps male harassment (Rivera and Andres 2002).

Perhaps most significantly though, when mated to a heterospecific, female fitness can be largely restored by mating with a conspecific. This effect seems to be independent of mating order (although we note that mating failure and oviposition failure appeared to be more common when the first mating was with a heterospecific). As mentioned above, crosses with *L. simulans* showed significantly reduced hatching success and egg number. This might be due to cryptic female choice, whereby females reduce egg laying when mated to incompatible or poor quality mates (Markow 1997). The reduction in egg number was more apparent when we measured female production across her lifespan, as differences in egg production between females mated to conspecifics and those mated to heterospecifics were more apparent later in life (Figure 4). However, reduced hatching success was seen in the heterospecific crosses at all stages of the females’ life (Figure 5). Although hybridization is clearly possible between the 2 species, little is known about the viability or fitness of these hybrids, although F1s and F2s can be produced (Evans et al. forthcoming). Most notably perhaps, we currently have no information about Haldane’s rule (which would predict loss of viability or fertility in male hybrids in this cross, as males are the heterogametic sex: Coyne and Orr 2004). We cannot tell from our results if reduced hatching is a result of females not using heterospecific sperm, as seen in flour beetles (Fricke and Arnqvist 2004) and several *Drosophila* species (Markow 1997), or simply a result of incomplete postmating reproductive isolation. However, mixed species pairings were frequently observed throughout the duration of the experiment and even when infertile females were excluded from the analysis, mean nymph production was lower in the *L. simulans* treatments, suggesting that this effect is not merely due to a failure to mate.

One aspect of heterospecific matings that may be important in determining the extent of sperm transfer is copulation duration. In both *L. equestris* and *L. simulans*, copulations must typically exceed 1h for sperm transfer to take place (Tadler 1999; Tadler et al. 1999). Copulation duration is assumed to be largely under male control (but see Sillen-Tullberg 1985 for potential mechanisms by which females may influence copulation duration), and male seed bugs have claspers with which they grasp females during matings. Variation in these claspers is one of the key features by which *L. equestris* and *L. simulans* can be distinguished from one another (Deckert 1985), and this variation may also affect the ability of males to remain in copula with females for long periods of time. If this is indeed the case then it may result in smaller amounts of sperm being transferred during heterospecific matings, leading to the lower number of fertilized eggs observed.

On the other hand, it may be that the genitalia of *L. simulans* are less efficient at transferring sperm. The reproductive anatomy of *Lygaeus* genitalia is complex, and we know there is stabilizing selection on intromittent organ length in intraspecific copulations in both *L*. *simulans* and *L*. *equestris* (Dougherty et al. 2015). Female cryptic choice may also play a role here, with the muscular valve between the spermathecal duct and the spermatheca possibly enabling the female to control which sperm is stored (Gschwentner and Tadler 2000). These potential postcopulatory mechanisms are likely to have evolved due to the limited precopulatory choice and the need for close contact to assess compatibility (Bretman et al. 2009; Burdfield-Steel et al. 2013; Dougherty and Shuker 2014). The cost of hybridization is high, yet *L. equestris* females do not completely reject matings with *L. simulans* males. Hence, female cryptic choice and sperm competition enable the female to reduce these costs without engaging in potentially damaging, precopulatory struggles with unsuitable males.

Other mechanisms that may be influencing female fecundity include physical damage caused by mismatched genital morphology during mating (Rönn et al. 2007) and the presence of male seminal proteins. Many species have been found to have nonsperm components of the seminal fluid or package that can influence female physiology and behavior (Chapman et al. 1995; Wigby et al. 2009; Perry et al. 2013). Although little is known about the components of seminal fluid in the Lygaeidae, males of 1 species (*Togo hemipterus*) have been found to influence female refractory period via accessory glad substances (Himuro and Fujisaki 2008). Given that such male adaptations usually coevolve alongside female resistance to the effects of these compounds within populations (Holland and Rice 1999; Andrés and Arnqvist 2001; Rönn et al. 2007), there is the potential for heterospecific male seminal compounds to negatively affect *L. equestris* females. Additionally, any compounds that have evolved to reduce the success of rival sperm, due to the risk of sperm competition in these species, may also impede the survival or fertilization success of heterospecific sperm within the female tract.

Conspecific sperm precedence has been reported in a number of studies (Price et al. 2000; Fricke and Arnqvist 2004). For example, in ﬂour beetles (*Tribolium spp*), cryptic homogamy occurred despite no obvious costs of hybridization, with conspecific males achieving a greater share of paternity (Fricke and Arnqvist 2004). In *Drosophila simulans*, prefertilization barriers obstruct heterospecific sperm, again resulting in conspecific sperm precedence (Price et al. 2000). Not only does conspecific seminal fluid incapacitate sperm from heterospecific males, it also physically displaces it from the reproductive tract (Price et al. 2000). Perhaps the most convincing evidence is in field crickets, where in a similar study to this current one, conspecific sperm was preferentially stored in the spermatheca, thus increasing the probability of intraspecific fertilization (Tyler et al. 2013). The biasing of fertilization can also occur during intraspecific multiple matings, for example, in copulations between different races of alpine grasshopper, in which a higher proportion of offspring from virgin females were sired by males from the same race as the focal female (*Podisma pedestris*: Hewitt et al. 1989).

It is important to note that we did not directly observe matings in the majority of pairings during these experiments. Thus, it is possible both that matings failed to occur during the 24-h period that males and females were housed together, or indeed that multiple matings occurred and that this varied between conspecific and heterospecific matings, for instance if matings take longer to initiate in heterospecific crosses. However, a recent study has found little variation in latency to mate within- and among-populations of *L*. *equestris* and *L*. *simulans* (Evans et al. forthcoming). Unsurprisingly, there is premating reproductive isolation between the 2 species, but it is asymmetric, with male *L*. *simulans* able to mate with female *L*. *equestris* (the direction of cross used in the current study), while male *L*. *equestris* rarely mated with female *L*. *simulans* (Evans et al. forthcoming).

Indeed, females restrict oviposition when virgin (Shuker et al. 2006) and so the hatching success data (number of nymphs produced) are unlikely to be influenced by failure to copulate. Among the heterospecific crosses, even if there is greater failure to mate, it is unlikely to be the sole factor influencing fitness. This is because amongst the females who had only had the opportunity to mate with *L. simulans* males, those females that did produce fertile eggs, and so must have mated, still had a much lower hatching success than females who had mated with conspecifics as well. On the other hand, if we have underestimated the number of matings amongst pairs (i.e., more multiple mating, but not more “true” polyandry), we should perhaps expect to see greater benefits when females have the opportunity to be polyandrous, but clearly this is not the pattern we see in the data. One final possibility is that heterospecific copulations may have been terminated more often, and thus be of shorter duration on average, than conspecific pairings, perhaps due to genitalia misalignments or other morphological mismatches. If this is the case then it is possible that this, and not postmating incompatibilities, may be driving the lower fitness of the females mated to heterospecifics. However, personal observations did not detect any striking differences in copulation duration between conspecific and heterospecific pairings, making it unlikely that they could be solely responsible for the dramatic reduction in hatching success seen in females mated only to *L. simulans*.

Finally, in our final experiment we found no evidence that CHCs played an important role in identifying compatible individuals or in facilitating the biasing of fertilization. Although we have some evidence that CHCs do influence precopulatory choice, the effects are not strong and the results presented here clearly show that heterospecific matings occur fairly readily among unmanipulated individuals (see also Shuker et al. 2015 for a broader survey of heterospecific matings in lygaeids).

In summary, inbreeding avoidance does not seem to be the driving cause of polyandry in the seed bug *L. equestris*. Our data thus fit with the emerging picture across a range of species that suggests that genetic compatibility tends to be a weak driver of polyandry (Simmons 2005; Slatyer et al. 2012). However, in areas where it co-occurs with closely related species such as *L. simulans*, multiple mating may allow females to avoid the costs associated with hybridization. The mechanism by which the rescue of fertility occurs remains unclear as manipulation of a cue that may be used by the females to assess male species or quality had no effect on either nymph or egg production.

## SUPPLEMENTARY MATERIAL

Supplementary material can be found at http://www.beheco.oxfordjournals.org/

## FUNDING

E.R.B.-S. was supported by a Natural Environment Research Council (NERC) studentship, and D.M.S. was in part supported by a NERC Advanced Fellowship (NE/D009979/2).

## Supplementary Material

Supplementary Data
