# Supplementary material for: Do the benefits of polyandry scale with outbreeding?
Source: Behav Ecol. 2015 Jul 1;26(5):1423–31. doi: 10.1093/beheco/arv103 (PMC4568444; doi:10.1093/beheco/arv103)
Supplement: Supplementary Data [file supp_26_5_1423__index.html]

Do the benefits of polyandry scale with outbreeding? — Do the benefits of polyandry scale with outbreeding? — Supplementary Data 

# Do the benefits of polyandry scale with outbreeding?

## Supplementary Data

Data files

- Supplementary Data - Supplementary Data
- Supplementary Data - Supplementary Data
- Supplementary Data - Supplementary Data
