# Supplementary figures and images for: Do the benefits of polyandry scale with outbreeding?
Source: Behav Ecol. 2015 Jul 1;26(5):1423–31. doi: 10.1093/beheco/arv103 (PMC4568444; doi:10.1093/beheco/arv103)

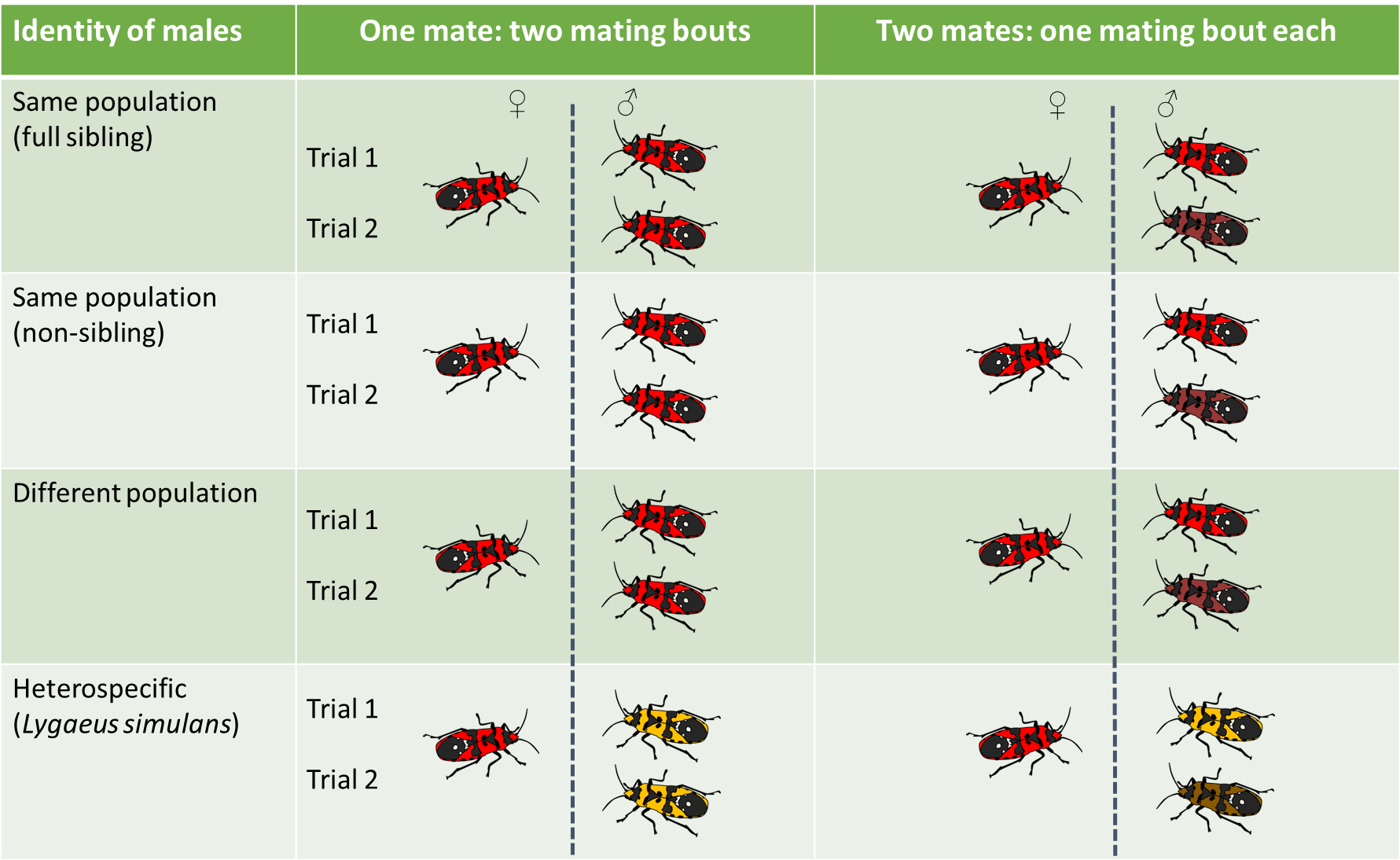

Supplement: Supplementary Data [file supp_arv103_SupFig1.tif]

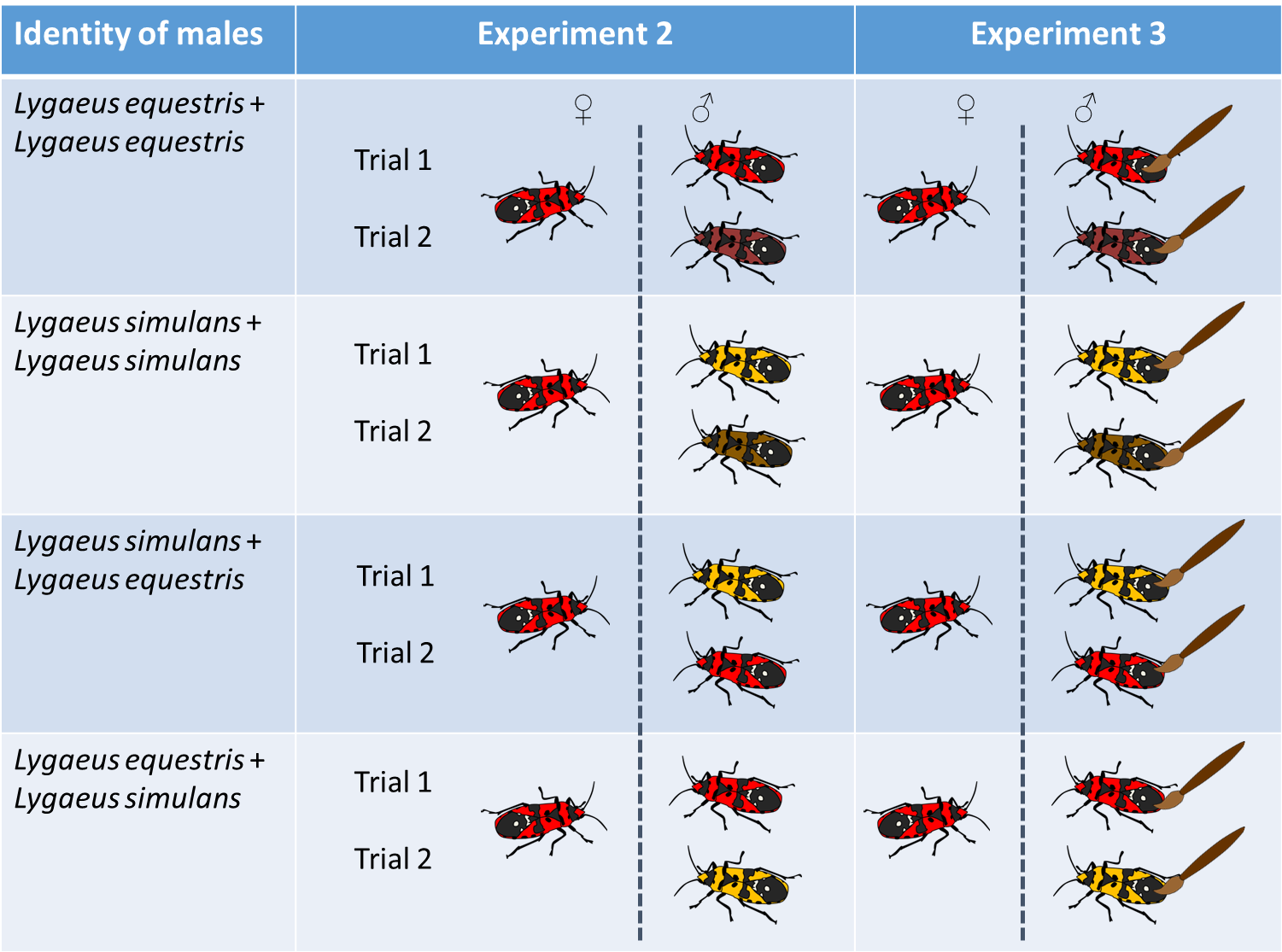

Supplement: Supplementary Data [file supp_arv103_SupFig2.tif]
