# Supplementary material for: Do the benefits of polyandry scale with outbreeding?
Source: Behav Ecol. 2015 Jul 1;26(5):1423–31. doi: 10.1093/beheco/arv103 (PMC4568444; doi:10.1093/beheco/arv103)
Supplement: Supplementary Data [file supp_arv103_Supplementary_Figure_Legends.docx]

Supplementary Figure Legends

Supplementary Figure 1. Diagram showing the design of experiment 1. Females (on the left) were paired twice with either one or two males from one of the four treatments. Red bugs indicate *Lygaeus equestris* while yellowbugs indicate *Lygaeus simulans*.

Supplementary Figure 2. Diagram showing the design of experiments 2 and 3. Females (on the left) were paired with two males according to the four treatments. Red bugs indicate *Lygaeus equestris* while yellow bugs indicate *Lygaeus simulans*. The brush indicated that males were washed with hexane prior to being introduced to the female.
